# Recipient obesity as a risk factor in kidney transplantation

**DOI:** 10.1186/s12882-022-02668-z

**Published:** 2022-01-18

**Authors:** Uwe Scheuermann, Jonas Babel, Uta-Carolin Pietsch, Antje Weimann, Orestis Lyros, Katrin Semmling, Hans-Michael Hau, Daniel Seehofer, Sebastian Rademacher, Robert Sucher

**Affiliations:** 1 Department of Visceral, Transplantation, Vascular and Thoracic Surgery, University Hospital of Leipzig, Liebigstrasse 20, 04103 Leipzig, Germany; 2 Department of Anaesthesiology and Intensive Care Medicine, University Hospital of Leipzig, Leipzig, Germany; 3 Department of Visceral, Thoracic and Vascular Surgery, University Hospital and Faculty of Medicine Carl Gustav Carus, Dresden University of Technology, Dresden, Germany

**Keywords:** Kidney transplantation, Obesity, Body mass index, Outcome, Survival

## Abstract

**Background:**

The aim of the study was to investigate the effect of recipient obesity on the short- and long-term outcomes of patients undergoing primary kidney transplantation (KT).

**Patients and methods:**

A total of 578 patients receiving primary KT in our department between 1993 and 2017 were included in the study. Patients were divided according to their body mass index (BMI) into normal weight (BMI 18.5–24.9 kg/m^2^; N = 304), overweight (BMI 25–29.9 kg/m^2^; N = 205) and obese (BMI ≥ 30 kg/m^2^; N = 69) groups. Their clinicopathological characteristics, outcomes, and survival rates were analyzed retrospectively.

**Results:**

Obesity was associated with an increased rate of surgical complications such as wound infection (*P* < 0.001), fascial dehiscence (*P* = 0.023), and lymphoceles (*P* = 0.010). Furthermore, the hospital stay duration was significantly longer in the groups with obese patients compared to normal weight and overweight patients (normal weight: 22 days, overweight: 25 days, and obese: 33 days, respectively; *P* < 0.001). Multivariate analysis showed that recipient obesity (BMI ≥ 30) was an independent prognostic factor for delayed graft function (DGF) (OR 2.400; 95% CI, 1.365–4.219; *P* = 0.002) and postoperative surgical complications (OR 2.514; 95% CI, 1.230–5.136; *P* = 0.011). The mean death-censored graft survival was significantly lower in obese patients (normal weight: 16.3 ± 0.6 years, overweight: 16.3 ± 0.8 years, obese 10.8 ± 1.5 years, respectively; *P* = 0.001). However, when using the Cox proportional hazards model, the association between recipient obesity and death-censored renal graft failure disappeared, after adjustment for important covariates, whereas the principal independent predictors of graft loss were recipient diabetes mellitus and hypertension and kidneys from donors with expanded donor criteria.

**Conclusion:**

In conclusion, obesity increases the risk of DGF and post-operative surgical complications after primary KT. Appropriate risk-adapted information concerning this must be provided to such patients before KT. Furthermore, obesity-typical concomitant diseases seem to negatively influence graft survival and need to be considered after the transplantation of obese patients.

## Introduction

Kidney transplantation (KT) is the treatment of choice in patients with end-stage renal disease (ESRD) and it improves both patient survival and recipients quality of life compared to chronic dialysis treatment [1–3].

Due to an aging society and changes in lifestyle – characterized by excessive calorie intake and a lack of physical activity – the percentage of overweight and obese patients has steadily increased in recent decades. According to the current Organisation for Economic Co-operation and Development (OECD) data, 60% of the population in Germany aged 15 years and older are overweight or obese [4]. Additionally, as obesity itself promotes ESRD, the proportion of obese renal transplant candidates is consequently increasing [5–9].

In general, surgery in obese patients is associated with a prolonged operative time and a higher risk for complications such as increased intraoperative blood loss and wound infections [10–13]. And higher prevalence of comorbidities in obese patients—such as cardiovascular disease, diabetes mellitus and hypertension – could jeopardize the allograft. Therefore, transplantation in obese recipients is still discussed controversially.

Thus, the current study sought to analyze the short- and long-term outcomes of obese ESRD patients undergoing primary KT.

## Patients and methods

### Data collection and study population

Medical data from all adult patients (≥ 18 years of age) who underwent initial living or deceased donor kidney transplantation (KT) at the University Hospital of Leipzig between October 1993 and December 2017 were retrospectively analyzed. Our data source comprised a prospectively collected electronic database. Patients undergoing multi-organ (combined) transplants or re-transplants, underweight patients (BMI < 18.5 kg/m^2^), and patients with missing (incomplete) data were excluded from the study. Follow-up data were collected until March 2020.

The characteristics of the study population included donor and recipient age, gender, and body mass index (BMI, weight in kg/ height in m^2^), donor cause of death, duration of dialysis, time on the waiting list, and Criteria of Expanded Criteria Donors (ECD). Peri- and post-transplant data included information on the number of human leukocyte antigen (HLA) -A, B, and DR mismatches (0–6), last pretransplant panel reactive antibody (PRA) levels, cold (CIT) and warm ischemia time (WIT) of the grafts, duration of operation, as well as immunosuppressive therapy. CIT is defined as the time that the organ spent in cold preservation solution after removal from the donor, while WIT is the time from cross-clamping until cold perfusion, plus the time of implantation (organ out of ice until reperfusion).

### Outcome measures

The outcome data included initial non-function (INF), biopsy-proven or clinically suspected episodes of acute rejection (in the first year after KT), delayed graft function (DGF), intra- and post-operative complications, date of graft failure, and patient death. INF was defined as dialysis dependence or creatinine clearance ≤ 20 mL/ min at three months post-transplant. Rejection episodes were histologically proved and DGF was defined as the need for dialysis in the first week following transplantation [14]. Post-operative complications occurring during the first three months after transplantation were analyzed. Complications included delayed wound healing, wound infection, urine leak, bleeding, development of hematoma, and lymphoceles. The Clavien-Dindo classification was used for morbidity assessment, and major morbidity was defined as being Clavien Dindo 3b or greater [15]. New-onset diabetes after transplantation (NODAT) was defined as the need for insulin or oral hypoglycemic drugs. Graft failure was defined as a return to dialysis or re-transplantation. Post-operative deaths included all deaths occurring within 30 days after surgery.

### Body mass index

Recipient body mass index (BMI) was calculated based on the formula: weight (kg)/ [height (m)^2^], from height and weight recorded at the time of transplantation. Patients were classified as normal weight (BMI 18.5–24.9 kg/m^2^), overweight (BMI 25–29.9 kg/m^2^), or obese (BMI ≥ 30 kg/m^2^) according to guidelines of the World Health Organization (WHO) [16].

### Glomerular filtration rate

Using serum creatinine levels, the estimated glomerular filtration rate (eGFR) was calculated using the Chronic Kidney Disease Epidemiology Collaboration (CKD-EPI) equation (mL/ min/ 1.73 m^2^ of standard body surface area (BSA)) [17]. To reduce errors induced by indexing the glomerular filtration rate for body surface area, the GFR was adjusted to the individual patient body surface area (eGFR x individual BSA [m^2^] / 1,73 m^2^ standard BSA = mL/ min) [18, 19].

### Standard and expanded criteria donors

The standard criteria donor (SCD) was defined as a donor who is under 50 years of age and suffered brain death from any cause. Criteria of Expanded Criteria Donors (ECD) kidneys are sourced from donors over 60 years of age or donors between 50 and 59 years of age with at least two of the following three criteria: cerebrovascular death, arterial hypertension, and a donor serum creatinine level > 1.5 mg/dl [20].

### Organ procurement and transplantation

The kidney grafts were procured according to the guidelines provided by Eurotransplant (ET) and transplanted into the iliacal fossa. Deceased donor kidneys were flushed in situ with cold HTK (histidine-tryptophan-ketoglutarate) solution and explanted. In living-related donation, kidneys were flushed with HTK after donor nephrectomy. For static cold storage, all grafts were immersed in HTK solution at 4 °C [21, 22]. The ureter was implanted into the bladder according to the Lich-Gregoir technique using a double J intra-ureteral splint [23, 24].

### Immunosuppression

The initial immunosuppressive therapy comprised an induction therapy with the interleukin-2 receptor antagonists (daclizumab [withdrawn from the market in 2018] or basiliximab) or antithymocyte globulin, followed by triple maintenance immunosuppression comprising calcineurin inhibitors (tacrolimus or cyclosporine), and/or mTOR inhibitors (everolimus or sirolimus), antimetabolites (azathioprine or mycophenolate mofetil), and tapered steroids (prednisolone). A rapid steroid-tapering regimen was applied in all our patients, starting with 500 mg methylprednisolone intraoperatively to reach a dose of 25 mg prednisolone at the end of the first week after transplantation. Further reduction intended a daily maintenance dose of 5 mg. Whenever possible, steroids were rapidly withdrawn and discontinued at the end of the first year post-transplant.

### Statistical analysis

For comparison between the groups, the appropriate statistical significance test, including Student’s t-test, the chi-squared test, analysis of variance (ANOVA), the Kruskal–Wallis test, and the Wilcoxon–Mann–Whitney test was used. Univariate and multivariate logistic regression analyses were used to evaluate the association between independent variables and binary outcomes of allograft function, and multivariate Cox proportional hazard analysis was applied to assess independent predictors of kidney graft failure. Prior to the regression analysis, post-operative complications were summarized into three groups: vascular (deep vein thrombosis, arterial or venous occlusion, secondary bleeding/hematoma), urological surgical (urine leakage), and surgical complications (wound infection, fascial dehiscence, lymphoceles). For the multivariate analyses, we used a forward stepwise regression model including only clinically relevant variables and those presenting P < 0.05 in univariate analysis. Survival rates were calculated using the Kaplan–Meier analysis, and the log-rank test was applied to test statistical significance. Graft survival was calculated as the time from initial transplant to graft failure (re-start of dialysis), uncensored for recipient death or censoring for death with a functioning graft. Patient survival was defined as the time from transplant to patient death, censoring for patients still alive at the time of analysis. If a recipient was alive or lost to follow-up at the time of the last contact, then survival time was censored at the time of the last contact. SPSS software, version 21.0 (SPSS Inc., Chicago, Illinois, USA) and Graphpad Prism software, version 9.2.0 (Graph-Pad Software Inc., La Jolla, CA) were used for statistical analysis and graphs. A P value < 0.05 was considered as statistically significant. Baseline data are presented as median values with the standard deviation (SD).

## Results

### Baseline characteristics

**Fig. 1:**
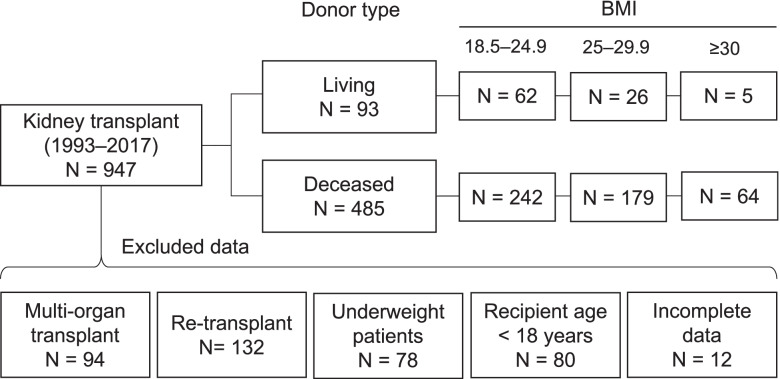
Flow chart illustrating the inclusion and exclusion criteria of patients in the study. BMI, body mass index. Category numbers do not sum up due to overlap between exclusion criteria

Between October 1993 and December 2017, 947 kidney transplants (KT) were performed in our department. A total of 578 patients could be included in the analysis, with 304 normal weight (52.6%), 205 overweight (35.5%), and 69 obese (11.9%) patients (Fig. 1).

The groups were similar in most of their baseline characteristics (Table 1). Median follow up was 5.8 ± 6.8 years. The proportion of obese patients significantly increased over the observation period (1993–2001: 7.0%, 2002–2009: 12.9%, and 2010–2017: 15.7%, respectively; *P* = 0.011). A higher BMI was associated with a significantly longer duration of surgery (*P* < 0.001); however, no significant differences between the groups were found for cold and warm ischemia time or intraoperative complications.

**Table 1:** Donor, recipient, and transplant characteristics

Variables	Body mass index			*P*-value
	18.5–24.9 (N = 304)	25–29.9 (N = 205)	≥ 30 (N = 69)	
Recipient				
Age, years	49.5 ± 14.2	55.9 ± 11.9	59.6 ± 12.2	< 0.001
Gender, male/ female (%)	182 (59.9)/ 122 (40.1)	135 (65.9)/ 70 (34.1)	46 (66.7)/ 23 (33.3)	0.304
BMI, kg/m^2^	22.4 ± 1.6	27.1 ± 1.3	31.9 ± 2.4	< 0.001
Time on the waiting list, months	17.7 ± 28.6	18.4 ± 30.2	23.0 ± 30.3	0.574
Dialysis duration, months	47.3 ± 35.5	46.4 ± 36.2	55.6 ± 33.6	0.763
Cause of ESRD (%)				
Glomerulonephritis	153 (50.0)	67 (32.7)	17 (24.6)	< 0.001
Non-glomerulonephritis, cystic kidney disease/ interstitial nephritis/ diabetes mellitus/ others/ unknown	47 (15.5)/ 28 (9.2)/ 3 (1.0)/ 50 (16.4)/ 24 (7.9)	39 (19.0)/ 26 (12.7)/ 16 (7.8)/ 42 (20.4)/ 15 (7.3)	15 (21.7)/ 7 (10.1)/ 10 (14.6) /16 (23.2)/ 4 (5.8)	
Comorbidity (%)				
Diabetes mellitus	26 (8.6)	30 (14.6)	22 (31.9)	< 0.001
Hypertension	276 (90.8)	193 (94.1)	67 (97.1)	0.118
Coronary disease	35 (11.5)	38 (18.5)	12 (17.4)	0.072
PVD	11 (3.6)	16 (7.8)	6 (8.7)	0.071
Donor				
Age, years	51 ± 15.6	53 ± 17.3	56 ± 16.4	0.069
Gender, male/ female (%)	165 (54.3)/ 139 (45.7)	114 (55.6)/ 91 (44.4)	41 (59.4)/ 28 (40.6)	0.737
BMI, kg/m^2^	25.1 ± 4.3	25.0 ± 3.7	24.4 ± 3.0	0.200
Organ quality, excellent/ good/ acceptable/ unknown (%)	14 (4.6)/ 216 (71.1)/ 10 (3.3)/ 64 (21.1)	17 (8.3)/ 136 (66.3)/ 11 (5.4)/ 41 (20.0)	5 (7.2)/ 50 (72.5)/ 4 (5.8)/ 10 (14.5)	0.329
Comorbidity (%)				
Diabetes mellitus	22 (7.2)	13 (6.3)	5 (7.2)	0.921
Hypertension	83 (27.3)	71 (34.6)	25 (36.2)	0.129
Donor type, LD/ SCD/ ECD (%)	62 (20.4)/ 146 (48.0)/ 96 (31.6)	26 (12.7)/ 100 (48.8)/ 79 (38.5)	5 (7.2)/ 32 (46.4)/ 32 (46.4)	0.014
Cause of death DD (%)				
CVA	125 (51.7)	92 (51.4)	35 (54.7)	0.571
Non-CVA, anoxia/ ischemia/ polytrauma/ others	18 (7.4)/ 28 (11.6)/ 38 (15.7)/ 8 (3.3)	18 (10.1)/ 16 (8.9)/ 25 (14.0)/ 13 (7.3)	7 (10.9)/ 4 (6.3)/ 9 (14.1)/ 3 (4.7)	
Transplant				
Transplant era (%)				
1993–2001/ 2002–2009/ 2010–2017	108 (35.5)/ 105 (34.5)/ 91 (29.9)	64 (31.2)/ 71 (34.6)/ 70 (34.1)	13 (18.8)/ 26 (37.7)/ 30 (43.5)	0.079
HLA mismatches ≥ 3 (%)	143 (47.0)	108 (52.7)	40 (58.0)	0.271
PRA (%)	50 (16.4)	29 (14.1)	10 (14.5)	0.753
Gender mismatch	153 (50.3)	109 (53.29	31 (44.9)	0.462
CIT, hours	11.5 ± 7.1	10.6 ± 6.6	11.4 ± 5.7	0.999
WIT, minutes	40 ± 19.9	40 ± 15.8	45 ± 23.8	0.312
Induction therapy, ATG/ ILR2-RA	15 (4.9)/ 101 (33.2)	7 (3.4)/ 68 (33.2)	2 (2.9)/ 26 (37.7)	0.582
Intra-operative complications (%)				
Bleeding	11 (3.6)	10 (4.9)	3 (4.3)	0.780
Thrombosis artery	8 (2.6)	6 (2.9)	3 (4.3)	0.748
Thrombosis vein	4 (1.3)	2 (1.0)	1 (1.4)	0.925
Hyperacute rejection	3 (1.0)	0	0	0.257
Duration of surgery, minutes	172 ± 46.0	179 ± 53.2	194 ± 64.8	< 0.001
Immunosuppression (%)				
CNI, Tac/ CsA	170 (55.9)/ 128 (42.1)	122 (59.5)/ 75 (36.6)	47 (68.1)/ 22 (31.9)	0.400
mTOR inhibitor, Ever/ Siro	1 (0.3)/ 8 (2.6)	0/ 4 (2.0)	1 (1.4)/ 1(1.4)	0.471
CNI + mTOR inhibitor	9 (3.0)	4 (2.0)	2 (2.9)	0.788
AM drug, AZA/ MMF	23 (7.6)/ 262 (86.2)	8 (3.9)/ 180 (87.8)	3 (4.3)/ 63 (91.3)	0.502
Steroids, prednisolone	295 (96.7)	191 (93.2)	69 (100.0)	0.134
Follow-up, years	7.6 ± 6.0	6.7 ± 5.9	5.5 ± 4.3	0.005

Data are shown as median ± SD

*AM* Antimetabolite, *Aza* Azathioprin, *ATG* Anti-thymocyte globulin, *BMI* Body mass index, *CNI* Calcineurin inhibitor, *CsA* Ciclosporin A, *CVA* Cerebrovascular accident, *CIT* Cold ischemia time, *DD* Deceased donor, *ECD* Expanded criteria donor, *ESRD* End-stage renal disease, *Ever* Everolimus, *HLA* Human leukocyte antigen-A, B, and DR, *IL2-RA* Interleukin-2 receptor antagonist, *LD* Living donor, *MMF* Mycofenolate mofetil, *mTOR* Mechanistic target of rapamycin, *PVD* Peripheral vascular disease*, PRA *Panel reactive antibody, *SCD* Standard criteria donor, *Siro* Sirolimus, *WIT* Warm ischemia time

### Outcome

The analysis of post-operative outcome parameters is shown in Table 2. In the overall study population, 32 kidneys lost their function in the first three months (initial non-function, INF) (normal weight, over-weight, and obese KT recipients: 11, 16, and 5, respectively, *P* = 0.108), whereas permanent lack of graft function from the time of transplantation (primary non-function, PNF) was observed in five cases (normal weight, over-weight, and obese KT recipients: 2, 3, and 0, respectively, *P* = 0.447). No cases of PNF or INF were reported in kidney grafts after living donation.

**Table 2:** Post-operative outcome parameters and immunosuppression after deceased donor kidney transplantation

Variables	Body mass index			*P*-value
	18.5–24.9 (N = 304)	25–29.9 (N = 205)	≥ 30 (N = 69)	
Outcome Parameters				
Surgical				
RBC substitution (%)	83 (27.3)	55 (26.8)	17 (24.6)	0.903
FFP substitution (%)	25 (8.2)	15 (7.3)	4 (5.8)	0.775
Time on ICU, days	5 ± 5.0	5 ± 6.0	5 ± 14.6	0.079
Post-operative complications (%)				
Deep vein thrombosis	3 (1.0)	4 (2.0)	2 (2.9)	0.435
Occlusion or thrombosis				
Renal artery	5 (1.3)	4 (2.0)	2 (2.9)	0.631
Renal vein	8 (2.6)	3 (1.5)	1 (1.4)	0.615
Pulmonary embolism	2 (0.7)	1 (0.5)	0	0.788
Secondary bleeding/ hematoma	62 (20.4)	39 (19.0)	16 (23.2)	0.754
Wound infection	48 (15.8)	45 (22.0)	30 (43.5)	< 0.001
Fascial dehiscence	1 (0.3)	5 (2.4)	3 (4.3)	0.023
Urine leakage	12 (4.0)	3 (1.5)	2 (2.9)	0.266
Lymphocele	37 (12.2)	19 (9.3)	16 (23.2)	0.010
Clavien-Dindo ≥ 3b (%)	67 (22.0)	51 (24.9)	26 (37.7)	0.025
Renal				
INF (%)	11 (3.6)	16 (7.8)	5 (7.2)	0.108
DGF (%)	72 (23.7)	47 (22.9)	32 (46.4)	< 0.001
Acute rejection, (%)	95 (31.3)	66 (32.2)	23 (33.3)	0.718
GFR (mL/ min)				
POD7	36.5 ± 42.5	45.5 ± 40.6	18.1 ± 28.3	0.002
POD14	67.6 ± 46.8	62.0 ± 36.8	56.3 ± 35.6	0.041
POM1	82.5 ± 36.5	76.7 ± 37.9	78.9 ± 35.9	0.160
POM6	85.4 ± 32.3	80.9 ± 35.7	93.6 ± 33.9	0.704
Metabolic				
NODAT (%)	15 (4.9)	12 (5.8)	8 (11.6)	0.110
Hospitalisation, days	22 ± 16.1	25 ± 16.2	33 ± 21.5	< 0.001
Post-operative mortality 30 days (%)	3 (1.0)	3 (1.5)	0	0.579
Post-operative mortality 90 days (%)	6 (2.0)	5 (2.4)	0	0.436

Data are shown as median ± SD

*DGF* delayed graft function, *FFP* fresh frozen plasma, *GFR* glomerular filtration rate, *ICU* intensive care unit, *INF* initial non-function, *NODAT* new-onset diabetes mellitus after transplantation, *POD* post-operative day, *POM* post-operative month, *RBC* red blood cells

**Fig. 2:**
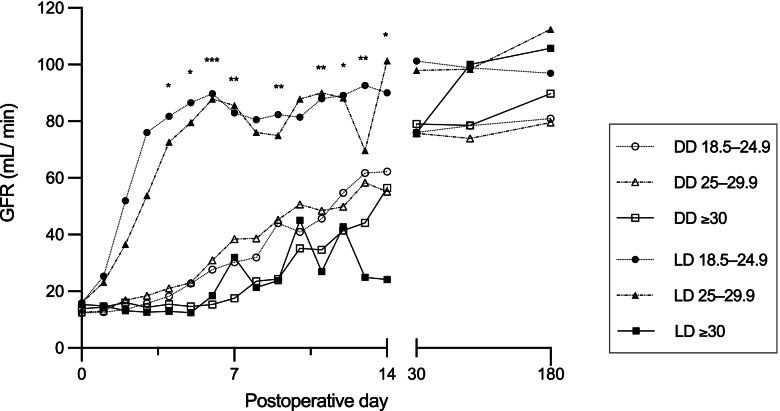
Post-operative glomerular filtration rate according to recipient body mass index and donor type. BMI, body mass index; DD, deceased donor; GFR, glomerular filtration rate; LD, living donor. * *P* < 0.05, ** *P* < 0.01, *** *P* < 0.001

Obese patients more frequently suffered from delayed graft function (DGF). These differences were most apparent in the subgroup of living donation (DGF normal weight, overweight, and obese, LD: 7 (11.3%), 2 (7.7%), and 3 (60.0%), respectively, *P* = 0.005; DD: 65 (26.9%), 45 (25.1%), and 29 (45.3%), respectively, *P* = 0.005). Fig. 2 shows glomerular filtration (GFR) rates among the groups within the first six months after KT.

The length of time spent in the intensive care unit after KT was comparable between the three groups (*P* = 0.079). However, the number of patients receiving ventilation post-transplant was significantly higher in the obese group (normal weight, over-weight, and obese KT recipients: 37 (12.2%), 36 (17.6%), and 16 (23.2%), respectively, *P* = 0.041), whereas the time of post-operative ventilation failed to show significance (normal weight, over-weight, and obese KT recipients: 2.0 ± 6.2 h, 2.0 ± 38.6 h, and 2.75 ± 33.1 h, respectively, *P* = 0.347).

Comparing Clavien-Dindo scores ≥ 3b, there was a significant difference in major complication rate between the groups (*P* = 0.025). Obesity was especially associated with an increased rate of surgical complications such as wound infection (*P* < 0.001), fascial dehiscence (*P* = 0.023), and lymphoceles (*P* = 0.010). Univariate analysis revealed that especially a recipient BMI ≥ 30 is significantly correlated with postoperative surgical complications and graft dysfunction (Table 3). This was underlined by a significantly longer hospital stay of obese patients compared to normal weight and overweight patients (normal weight, over-weight, and obese KT recipients: 22 days, 25 days, and 33 days, respectively; *P* < 0.001).

**Table 3:** Univariate analysis of recipient BMI and peri-operative kidney transplant outcome

Variables	Body mass index						
	18.5–24.9	25–29.9			≥ 30		
		Univariate analysis			Univariate analysis		
		OR	95% CI	P-value	OR	95% CI	*P*-value
Intraoperative complications							
Bleeding (1)	ref	1.366	0.569–3.278	0.485	1.211	0.329–4.461	0.774
Thrombosis artery or vein (1)	ref	0.988	0.397–2.461	0.980	1.106	0.304–4.030	0.879
Duration of surgery (1)	ref	1.391	0.960–2.016	0.081	2.765	1.489–5.134	0.001
Ventilation time (1)	ref	1.524	0.871–2.665	0.140	2.510	1.236–5.097	0.011
Time on ICU (1)	ref	0.756	0.528–1.082	0.126	1.082	0.632–1.851	0.773
Postoperative complications							
Vascular (1)	ref	0.897	0.586–1.374	0.671	1.339	0.747–2.402	0.327
Surgical (1)	ref	1.131	0.783–1.633	0.512	3.240	1.880–5.585	< 0.001
Urological surgical (1)	ref	0.361	0.101–1.297	0.118	0.726	0.159–3.322	0.680
Clavien-Dindo ≥ 3 (%)	ref	1.171	0.772–1.777	0.457	2.148	1.225–3.734	0.008
Hospitalisation (1)	ref	1.222	0.856–1.743	0.269	2.456	1.400–4.307	0.002
Renal outcome							
INF (1)	ref	2.232	1.014–4.914	0.046	2.092	0.702–6.234	0.185
DGF (1)	ref	0.946	0.622–1.440	0.796	2.827	1.640–4.875	< 0.001
Acute rejection, all (2)	ref	1.121	0.861–1.458	0.397	1.494	0.861–2.171	0.035
Graft failure (death-censored) (2)	ref	1.113	0.767–1.617	0.573	2.405	1.512–3.827	< 0.001
NODAT (1)	ref	1.198	0.549–2.615	0.650	2.527	1.026–6.224	0.044

Prior to regression analysis, post-operative complications were summarized into three groups: vascular (deep vein thrombosis, arterial or venous occlusion, secondary bleeding/ hematoma), urological surgical (urine leakage), and surgical complications (wound infection, fascial dehiscence, lymphoceles).1, Univariate log regression; 2, univariate cox regression; *95% CI* 95% confidence interval, *DGF* Delayed graft function, ICU Intensive care unit, *INF* Initial non-function, *NODAT* new-onset diabetes mellitus after transplantation, *OR* odds ratio, *Ref* Reference

In the group of normal-weight and overweight patients, there were five in-hospital deaths each, whereas no in-hospital deaths were reported in the obese group (P = 0.585). The causes of death included septic shock and multiple organ failure (N = 3), liver insufficiency (N = 1), and subdural hematoma (N = 1) in the normal weight group and septic shock (N = 1), endocarditis (N = 1), acute bleeding with cardiac arrest (N = 1) and acute heart failure (N = 2) in the overweight group, respectively.

In the overall study period, acute rejection was clinically suspected or histologically proven in 184 (31.8%) patients. In 149 cases, percutaneous kidney biopsies were performed and revealed acute rejection in 57 kidney allografts (38.3%), without showing any significant difference between the three groups (*P* = 0.918). However, an increase in the total number of treated rejection episodes per KT could be demonstrated among the groups, although these trends were also not statistically significant (normal weight: 8.1/ 100 graft years, overweight: 9.6/ 100 graft years, and obese: 17.7/ 100 graft years, respectively; *P* = 0.133) within the investigation period.

### Graft and patient survival

**Fig. 3:**
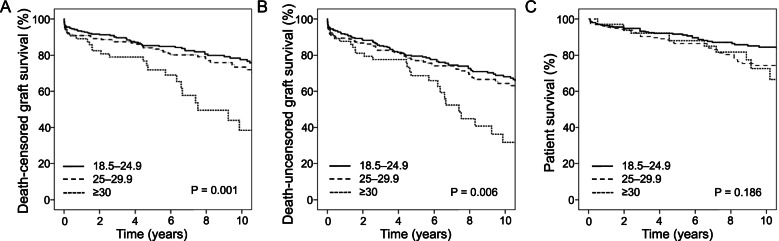
Graft and patient survival according to the body mass index. **A** Death-censored kidney graft survival, **B** death-uncensored kidney graft survival and **C** patient survival ten years after kidney transplantation

Figure 3 shows Kaplan–Meier survival curves of grafts and patients after KT according to their BMI. The one-, three-, five- and ten-year cumulative death-censored graft survival rates were 92%, 88%, 83%, and 72%, respectively. The mean cumulative death-censored graft survival was 15.9 ± 0.5 years. Mean death-censored graft survival was significantly lower in obese patients (normal weight: 16.3 ± 0.6 years, overweight: 16.3 ± 0.8 years, obese 10.8 ± 1.5 years, respectively; *P* = 0.001). After ten years of follow-up, graft survival was 39% in the obese group compared with 78% in the normal weight and 73% in the overweight group.

The one-, three-, five- and ten-year cumulative patient survival rates were 96%, 92%, 89%, and 79%, respectively. The mean patient survival was 18.7 ± 0.5 years. The one-, three-, five- and ten-year survival rates, as well as the mean patient survival, were comparable between the groups (mean patient survival: normal weight: 19.2 ± 0.6 years, overweight: 17.6 ± 0.7 years, and obese: 15.8 ± 1.5 years, respectively; *P* = 0.186).

The causes of graft loss are summarized in Table 4. The main reasons for graft failure were acute or chronic rejection (33.1%) and chronic allograft nephropathy (22.3%). Among the three BMI groups, the percentage of graft losses due to infection was significantly enhanced in obese KT recipients (6 (8.1%), 5 (10.4%), 9 (31.0%); *P* = 0.006). No statistical difference could be observed between the three groups in regard to graft loss due to rejection (29 (39.2%), 13 (27.1%), 7 (24.1%); *P* = 0.193).

**Table 4:** Causes of renal graft loss according to recipient body mass index. Category numbers do not sum up due to overlap between causes of graft failure (normal weight: N = 2, obese: N = 1)

Variables	Body mass index			*P*-value
	18.5–24.9 (N = 304)	25–29.9 (N = 205)	≥ 30 (N = 69)	
Cause of graft loss (%)				
Death	23 (7.6)	13 (6.3)	4 (5.8)	0.125
Graft failure	72 (23.7)	48 (23.4)	28 (40.6)	0.007
Cause of graft failure (%)				
Vascular	7 (9.5)	8 (16.7)	3 (10.3)	0.505
Acute or chronic rejection	29 (39.2)	13 (27.1)	7 (24.1)	0.193
Recurrence of renal disease	4 (5.4)	2 (4.2)	1 (3.4)	0.893
Chronic allograft nephropathy	19 (25.7)	8 (16.7)	6 (20.7)	0.452
Infection	6 (8.1)	5 (10.4)	9 (31.0)	0.006
Other	9 (12.2)	12 (25.0)	3 (10.3)	0.130

In the multivariate regression analysis, obesity (BMI ≥ 30) remained an independent predictor of DGF (OR 2.400; 95% CI, 1.365–4.219; *P* = 0.002) and postoperative surgical complications (OR 2.514; 95% CI, 1.230–5.136; *P* = 0.011) (Table 5). However, after adjusting for important covariates, obesity failed to be an independent predictor of decreased graft survival or acute rejection. Independent predictors of graft loss were recipient diabetes mellitus and hypertension and kidneys from donors with expanded donor criteria. Expanded criteria donor was the only independent predictor of acute rejection (treated acute rejection (OR 1.448; 95% CI, 1.131–1.852; *P* = 0.003), biopsy-proven acute rejection (OR 1.919; 95% CI, 1.236–2.978; *P* = 0.004)).

**Table 5:** Multivariate regression analysis of predictors of delayed graft function, surgical post-operative complications, rejection, and kidney graft loss after kidney transplantation

A								
Variables	Delayed graft function (1)				Surgical complications (1)			
	UVA	Multivariate analysis			UVA	Multivariate analysis		
	*P*-value	OR	95% CI	*P*-value	*P*-value	HR	95% CI	*P*-value
Recipient								
Age	0.001	1.535	1.003–2.350	0.048	0.040	NS	NS	NS
BMI								
18.5–24.9 vs. 25–29.9	0.796	0.821	0.530–1.273	0.379	0.512	1.061	0.631–1.784	0.823
18.5–24.9 vs. ≥ 30	< 0.001	2.400	1.365–4.219	0.002	< 0.001	2.441	1.182–5.038	0.016
Diabetes mellitus	0.179				0.075			
Hypertension	0.512				0.388			
Coronary disease	0.043	NS	NS	NS	0.202			
Donor								
Age	0.077				0.296			
Donor type, LD/ DD	0.002	2.118	1.091–4.112	0.027	0.071			
Cause of death, CVA/ non-CVA	0.960				0.457			
Diabetes mellitus	0.358				0.700			
Hypertension	0.013	NS	NS	NS	0.027	NS	NS	NS
ECD	0.001	1.538	1.016–2.328	0.042	0.194			
Transplant								
WIT	0.294				0.003	1.906	1.183–3.071	0.008
Duration of surgery	0.017	NS	NS	NS	0.380			
Acute rejection	N/A				0.005	2.006	1.084–3.713	0.027
B								
Variables	Rejection (treated) (2)			Graft failure (death-censored) (2)				
	UVA	Multivariate analysis			UVA	Multivariate analysis		
	*P*-value	HR	95% CI	*P*-value	*P*-value	HR	95% CI	*P*-value
Recipient								
Age	0.146				0.051			
BMI								
18.5–24.9 vs. 25–29.9	0.397	NS	NS	NS	0.573	NS	NS	NS
18.5–24.9 vs. ≥ 30	0.035	NS	NS	NS	< 0.001	NS	NS	NS
Diabetes mellitus	0.034	NS	NS	NS	0.001	1.709	1.067–2.736	0.026
Hypertension	0.053				< 0.001	2.565	1.514–4.344	0.001
Coronary disease	0.283				0.194			
Donor								
Age	0.004	NS	NS	NS	< 0.001	2.725	1.841–4.033	< 0.001
Donor type, LD/ DD	0.046	NS	NS	NS	0.004	NS	NS	NS
Cause of death, CVA/ non-CVA	0.191				0.031	NS	NS	NS
Diabetes mellitus	0.089				0.011	NS	NS	NS
Hypertension	0.182				< 0.001	NS	NS	NS
ECD	0.004	1.448	1.131–1.852	0.003	< 0.001	3.095	2.115–4.530	< 0.001
Transplant								
WIT	0.333				0.497			
Duration of surgery	0.144				0.375			
Acute rejection	N/A				< 0.001	2.709	1.830–4.011	< 0.001

Following variables were tested in univariate analysis but failed to show significancy: cold ischemia time, delayed graft function; donor BMI, donor cause of death (CVA versus non-CVA), donor comorbidity diabetes mellitus, HLA, human leukocyte antigen-A, B, and DR (0–2 versus ≥ 3); initial immunosuppression (calcineurin inhibitor versus mTOR inihibitor), induction therapy, initial non-function organ quality (excellent–good versus acceptable–poor), panel reactive antibodies, peripheral vascular disease; transplant era. 1, Univariate log regression; 2, univariate cox regression; *95% CI* 95% Confidence interval, *BMI* Body mass index, *CVA* Cerebrovascular accident*, DD* Deceased donor, *ECD* Expanded criteria donor, *HR* Hazard ratio, *LD* Living donor, *NS* Not significant, *OR* Odds ratio, *UVA* Univariate analysis, *WIT* Warm ischemia time

## Discussion

The proportion of obese patients receiving kidney transplantation (KT) has been increasing over the last decades [7–9] and is subsequently becoming one of the leading challenges for transplant surgeons. In the following, we will address the effect of obesity on the short- and long-term outcomes after living and deceased donor KT.

### Post-operative complications

In the current study, obese KT recipients were significantly more likely to experience surgical complications such as wound infections, fascial dehiscence, and lymphoceles, compared with normal weight and overweight KT recipients. In reviewing the literature, wound complications were among the most common post-operative complications in obese patients after KT [25], and in accordance with the present results, previous publications have demonstrated that lymphoceles occur more often in obese patients after KT [26–28]. It is assumed that poor vascularization and the reduced immune response of fat tissue lead to poorer wound healing in this group of patients. Furthermore, a larger wound area and technical challenges due to more difficult exposure result in longer operation times and thus in higher probabilities of wound infection in obese patients [10–13]. The length of hospital stays, which is a surrogate marker for post-operative complications, was also significantly prolonged in the group of obese patients. This is in line with the retrospective study from Gore et al. published in 2006 about 20,000 patients after living or deceased KT, which revealed an independent effect of recipient BMI on the length of hospital stay in the adjusted analysis [29].

Despite a higher rate of major postoperative complications, no post-operative deaths occurred in the group with obese kidney grafts recipients within the first three months after KT in our study cohort. Especially cardiovascular risk factors must be excluded prior to KT, as fatal cardiac events are a common cause of morbidity and death after KT in obese recipients [30–33]. Therefore, in our department, all patients routinely receive a cardiac stress test and, if necessary, a cardiac catheterization before enlisting for KT.

### Delayed graft function

Consistent with previous publications, we could identify recipient obesity as an independent risk factor of delayed graft function (DGF) in our cohort [25]. In a retrospective single-center study of 1,132 kidney transplant recipients, Weissenbacher et al. also showed that recipient BMI correlates with the incidence of DGF after deceased donor KT (recipient BMI: OR 1.087; 95% CI, 1.043–1.132; P < 0.0001) [34]. Additionally, a previously published retrospective multi-center study by Foucher et al. including 3,071 non-obese (BMI < 30) and 615 obese (BMI ≥ 30) recipients of kidney transplants confirmed this observation (recipient BMI: OR 1.89; 95% CI, 1.56–2.29; P < 0.0001) [33].

Although the pathogenesis of DGF has not yet been fully elucidated, it is thought to be a result of immunologically and ischemia-induced graft injury [14, 35]. In our study, neither the duration of surgery, nor the ischemia time of the graft reached statistical significance concerning DGF [29, 34, 36]. However, it should be noted that there is an additional risk of DGF in obese patients when receiving kidneys from cadaveric donation, which have an increased susceptibility to ischemic injury [29, 35–37]. Therefore, the percentage of DGF after deceased donor KT is inherently higher compared to living donor KT, as the prevalence of DGF ranges from 4 to 10% in patients after living KT, and between 2 and 50% in kidneys from brain-dead donors [35, 38]. In our study, the rate of DGF was remarkably high in the subgroup of obese patients receiving kidneys after living donation (60%). However, due to the low number of patients in this group (N = 5), a conclusive statement is not possible and further studies with larger patient number are needed.

Furthermore, it should be noted that in obese KT recipients, renal function was only reduced in the early postoperative period after transplantation. Six months after KT, eGFR were comparable between the three study groups, indicating a good graft function even in KT recipients with a higher BMI.

### Acute rejection episodes

A tendency towards a higher frequency of acute rejections per graft could be observed in the obese group of our patients. Meta-analysis showed that patients with a higher BMI have a significantly greater risk of acute rejection after KT [26]. Especially morbid obesity (BMI ≥ 35) demonstrated an association with acute rejection [29, 39]. A more difficult dosage adjustment, the maintenance of an appropriate level of immunosuppression, and a “state of chronic low-grade inflammation “ in obese patients could be reasons for the enhanced rate of acute rejection in this group [40–42]. In our analysis, due to low patient numbers (BMI ≥ 35, N = 8), we could not evaluate the effects of an extreme BMI on outcomes after kidney transplantation. Further studies are necessary to analyze the serum levels of immunosuppressants in obese KT recipients and, in conjunction with those from acute graft rejection.

### Graft survival

In our study, compared with normal weight or overweight KT recipients, graft survival in obese KT recipients was significantly reduced after five and ten years of observation. Several previous reports have shown that obesity itself confers a negative outcome on kidney graft survival [25, 31, 36, 39, 43–45]. However, after adjusting for important covariates, higher BMI was not an independent predictor for decreased graft survival in our analysis. Hence, the reasons for the increased risk of graft failure in overweight transplant recipients are most probably multifactorial. No correlation between the transplant era and patient outcome could be found in our data set. A higher prevalence of comorbidities in obese patients – such as cardiovascular disease, diabetes mellitus, and hypertension – could jeopardize the allograft, and an imbalance between donor and recipient weight might lead to renal hyperfiltration and graft injury [43]. In our study, obese patients were significantly older compared with non-obese patients at the time of KT and showed higher frequencies of secondary diseases of which the prevalence of diabetes mellitus and hypertension had an independent influence on all-cause graft failure. Especially since a new-onset diabetes mellitus is more often seen in obese patients after KT [33, 46, 47], this underlines the importance of strict follow-up care with intensive control of diabetes and high blood pressure to prevent or delay kidney graft failure.

### Limitations

There are some limiting factors in this study. First, the retrospective non-randomized design of the study and single-center effect should be mentioned. Second, the long investigation period restricted data evaluation, thereby making further controlled and prospective studies necessary. Third, we solely used BMI to define obesity in our study, which may not be an appropriate measure to characterize the nutritional status of a patient. Therefore, further studies, with a measurement of body fat distribution and muscle mass, and their association with the risk of morbidity and mortality in transplant recipients would be of interest. Fourth, the influence of weight loss or gain on the KT outcome and possibilities of bariatric surgery could be highlighted in future studies.

## Conclusion

In conclusion, data from our center indicate that obese patients after KT seem to suffer from surgical complications and DGF more frequently than non-obese patients. Therefore, appropriate risk-adapted information must be provided to these patient groups before KT. Furthermore, although obesity itself may not directly impair the survival of the kidney graft, secondary diseases such as diabetes mellitus and hypertension must be taken into consideration in obese KT recipients as they seem to negatively affect long-term graft survival.

## Data Availability

Our database contains highly sensible data which may provide insight in clinical and personnel information about our patients and lead to identification of these patients. Therefore, according to organizational restrictions and regulations these data cannot be made publicly available. However, the datasets used and/or analyzed during the current study are available from the corresponding author on reasonable request.

## Abbreviations

BMI: Body mass index
CIT: Cold ischemia time
CNI: Calcineurin inhibitor
CVA: Cerebrovascular accident
DD: Deceased donor
DGF: Delayed graft function
ECD: Expanded criteria donors
ESRD: End-stage renal disease
Ever: Everolimus
FFP: Fresh frozen plasma
GFR: Glomerular filtration rate
INF: Initial non-function
KT: Kidney transplantation
LD: Living donor
MMF: Mycophenolate mofetil
NODAT: New-onset diabetes after transplantation
POD: Post-operative day
POM: Post-operative month
PVD: Peripheral vascular disease
SD: Standard deviation
Siro: Sirolimus
WIT: Warm ischemia time
